# The size of the primary tumor and age at initial diagnosis are independent predictors of the metastatic behavior and survival of patients with *SDHB*-related pheochromocytoma and paraganglioma: a retrospective cohort study

**DOI:** 10.1186/1471-2407-14-523

**Published:** 2014-07-21

**Authors:** Jan Schovanek, Victoria Martucci, Robert Wesley, Tito Fojo, Jaydira del Rivero, Thanh Huynh, Karen Adams, Electron Kebebew, Zdenek Frysak, Constantine A Stratakis, Karel Pacak

**Affiliations:** 1 Program in Reproductive and Adult Endocrinology, Eunice Kennedy Shriver National Institute of Child Health & Human Development, NIH, Building 10, CRC, Room 1E-3140, 10 Center Drive MSC-1109, Bethesda, Maryland 20892-1109, USA; 2 Department of Internal Medicine III – Nephrology, Rheumatology and Endocrinology, Faculty of Medicine and Dentistry, Palacky University, Olomouc, Czech Republic; 3 Warren G. Magnuson Clinical Center, NIH, Bethesda, MD, USA; 4 Medical Oncology Branch, National Cancer Institute, NIH, Bethesda, MD, USA; 5 Endocrine Oncology Branch, National Cancer Institute, NIH, Bethesda, MD, USA; 6 Program on Developmental Endocrinology and Genetics, Eunice Kennedy Shriver National Institute of Child Health & Human Development, NIH, Bethesda, MD, USA

**Keywords:** Pheochromocytoma, Paraganglioma, Size, Age, SDHB, Metastatic, Survival

## Abstract

**Background:**

Succinate dehydrogenase subunit B (*SDHB)* mutations are associated with aggressive pheochromocytoma (PHEO)/paraganglioma (PGL) behavior, often resulting in metastatic disease and fatal outcomes. These tumors are often larger, extra-adrenal, and contain lower catecholamine concentrations than other hereditary PHEOs/PGLs. This study evaluated the size and age at diagnosis of primary *SDHB*-related PHEOs/PGLs as independent predictors of their metastatic behavior and outcome (survival).

**Methods:**

One hundred six patients with *SDHB* mutation-related PHEO/PGL were included in this retrospective study. The recorded largest diameters, locations, and patient ages at initial diagnosis of *SDHB*-related primary tumors were analyzed in the context of time to metastasis and patient survival.

**Results:**

First, the development of metastatic disease in patients with primary tumors ≥4.5 cm was significantly earlier than in patients with smaller tumors (P = 0.003). Second, patients with primary tumors larger than 5.5 cm also had worse overall survival than patients with smaller tumors (P = 0.008). Third, age at initial diagnosis was found to be an independent predictor of patient survival (PHEOs: P = 0.041; PGLs: P < 0.001). Fourth, we did not observe a significant difference in survival based on the specific *SDHB* mutations or patient sex.

**Conclusion:**

Receiver operating characteristic curves established 4.5 cm as the best value to dichotomize the primary *SDHB*-related PHEO/PGL in order to evaluate the development of metastatic disease and 5.5 cm as the best value for survival prediction. Subsequently, the size of the primary tumor was found as an age-independent predictor of patient survival and metastases development in PGL. In both PHEO and PGL, age at diagnosis was found to be a size-independent predictor of patient survival. No significant difference was found in metastases development or patient survival between males and females or among specific *SDHB* mutations. This data further extends and supports previous recommendations that carriers with *SDHB* mutations must undergo early and regular evaluations to detect PHEO/PGL in order to achieve the best clinical outcome.

## Background

According to the 2004 WHO classification of tumors, pheochromocytomas (PHEOs) arise from chromaffin cells of neural crest origin in the adrenal medulla. Closely related paragangliomas (PGLs) arise from cells of sympathetic or parasympathetic paraganglia [1]. These tumors synthesize catecholamines that are metabolized to metanephrines, which are preferentially used in the biochemical diagnosis of these tumors [2].

Mutations in succinate dehydrogenase subunit B (*SDHB)*, first described by the pioneering work of Astuti et al. in 2001 [3], have been linked to more aggressive tumor behavior, presenting with a higher metastatic rate than other PHEOs/PGLs [3-7]. The rate of metastasis of *SDHB*-related PHEOs/PGLs has been reported to be between 34% [8] and 71% [9], with a 5-year survival rate of 36% after the diagnosis of metastasis [5]. Other *SDHx* mutations have approximate metastatic rates as follows: SDHA, 0-14%; SDHC, rarely malignant; SDHD, <5% [10]. In addition, regardless of *SDHB* mutation status, tumor size has also been shown to be related to developing metastatic disease [11]. Recently, Eisenhofer and colleagues have described an increase in the likelihood of metastases in PHEOs from less than 6% for tumors smaller than 5 cm to over 50% in tumors larger than 10 cm; for PGLs, the rate of malignancy increases to over 80% for tumors larger than 9 cm. However, in their study, the genetic background was not considered [12].

While *SDHB* mutations are considered powerful predictors of malignancy, it is unclear why *SDHB*-related PHEOs/PGLs in particular are more aggressive, often metastatic, and ultimately fatal, even though some other hereditary PHEOs/PGLs are also pseudohypoxic and belong to the same Cluster I [13,14]. Some unique insights into the presentation and pathogenesis of these tumors have been published recently by Eisenhofer et al. [12,15], Loriot et al. [16], and Amar et al. [5,9]. These studies either confirmed or first showed that *SDHB*-related PHEOs/PGLs are most commonly extra-adrenal and larger at first presentation, with a characteristic noradrenergic and/or dopaminergic biochemical phenotype, as well as much lower catecholamine tumor concentrations than any other sporadic or hereditary PHEOs/PGLs. As a result of this lower catecholamine content, *SDHB* patients may initially present with only mild clinical symptoms that do not become worrisome until a sufficient amount of catecholamines is released, often in cases of already large primary tumors.

Of the unique *SDHB-*related PHEO/PGL characteristics described above, extra-adrenal location, age at initial presentation, size of the primary tumor, and elevated methoxytyramine levels were introduced and confirmed as risk factors for the metastatic behavior of PHEOs/PGLs [11,17-19]. Therefore, it has been recommended that patients with *SDHB*-related, large, or extra-adrenal PHEOs/PGLs should have more frequent and lifelong follow-up [20].

In the present study, we initially used receiver operating characteristic (ROC) curves to establish cut-off sizes for evaluation of the development of metastases and patient survival. We hypothesized that even in the presence of an *SDHB* mutation, smaller tumors would have a statistically significantly lower metastatic potential and longer patient survival than larger tumors. Subsequently we created Cox regression models aiming to establish whether those parameters could also be considered as independent predictors of PHEO/PGL metastatic behavior and patient outcome. The effect of having a specific *SDHB* gene mutation, adrenal or extra-adrenal tumor location, and their occurrence in males or females were also analyzed statistically. Finally, survival and metastatic potential parameters, including the presence of synchronous and metachronous metastases, were evaluated for both 5-year and overall survival.

## Methods

We performed a single center retrospective study, evaluating only patients with *SDHB*-related PHEOs/PGLs seen for evaluation or treatment at the National Institutes of Health (NIH), a referral center for these tumors. Imaging, surgical, and pathological study reports were carefully reviewed in order to collect the most accurate information about the patients. The follow-up data and information were collected based on patients’ regular follow-up visits at NIH or from patients’ relatives who reported their death. All patients provided written informed consent. The study was conducted at the NIH under Institutional Review Board (IRB)-approved protocol 00-CH-0093.

Metastases were confirmed either by surgery or by anatomical and PHEO/PGL-specific functional imaging studies. When there was evidence of lesions in areas where chromaffin cells are not present, these lesions were considered metastatic. For the purposes of the present study, when the metastases were observed together with a recurrent tumor, the tumor was marked as metastatic; recurrences were not evaluated. We use the term synchronous metastases to describe the occurrence of metastases discovered at initial diagnosis or within 6 months after the primary tumor diagnosis. Its complementary group, patients without synchronous metastases, was divided into those developing metastases after 6 months and those who did not develop any detectable metastases during the data collection period. The metastases developing after 6 months from initial diagnosis are characterized as metachronous [19]. The time to metastasis parameter is the interval between the initial diagnosis and the diagnosis of metastatic disease (defined as 0 for those with synchronous metastases).

For analyses of the effect of the size of the primary tumor for the development of metastases, we used the largest diameter size of 4.5 cm as a cutoff point, the optimal value (which maximized the sum of the sensitivity and specificity) based on ROC analysis; using this cutoff, the Area Under the Curve (AUC) = 0.782, sensitivity = 80.5%, specificity = 69%. For survival analysis, we dichotomized the patient cohort using the largest diameter size of 5.5 cm, the optimal cutoff point from ROC analysis for this endpoint (AUC = 0.663, sensitivity = 87.0%, specificity = 49.5%). This value divided the patients almost equally (Additional file 1: Figure S1). Alternatively, for some analyses the patients were divided into four nearly equal-sized groups according to the size of the primary tumor (≤4 cm, 4–6 cm, 6–9 cm, >9 cm). Analyses of the effects of various parameters on the time to metastases and for survival used Kaplan-Meier curves to graphically represent the results, with group comparisons based on the standard logrank test (to compare 2 groups), the trend version of it (to compare more than 2 groups that are ordered), or stratified versions of these (to adjust for a second parameter, such as PGL vs. PHEO). Survival analyses were reported either for total survival or for survival truncated at 5 years (i.e. anyone whose observation time was longer than 5 years was considered censored at 5 years); the same applied for “survival” analyses of time to metastases.

To analyze the mutual effects of age at diagnosis and the size of the primary tumor on survival, we used Cox regression models, with 5 ordered categories for age (defined by cutpoints at 20, 30, 40, 50 years old) and the aforementioned 4 ordered categories for size. Cox regression was also used to estimate the relative hazard rates for the 4 ordered size categories. For an alternative nonparametric, model-free estimate of the probability of death or metastases vs. tumor size, observations were divided into bins and the lowess smoother was applied to the proportions of outcomes in the bins. All survival results used death due to disease as the endpoint. All P-values were two-sided.

## Results

### Patient and tumor characteristics

**Figure 1:**
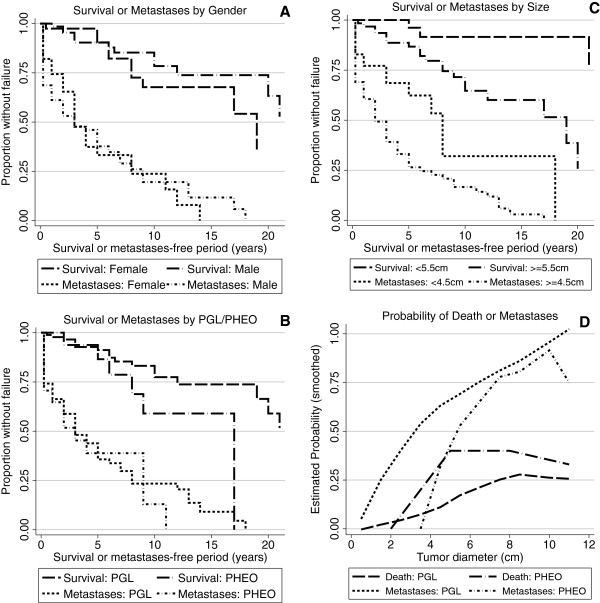
**Plots of survival and time to metastases were constructed based on patient characteristics.** Patients were separated based on gender **(Panel A)**, tumor type **(Panel B)**, primary tumor size **(Panel C)**, and linearly increasing primary tumor size **(Panel D)**.

One hundred six patients (39 females, 67 males) with *SDHB*-related PHEOs/PGLs from the *Eunice Kennedy Shriver* National Institute of Child Health & Human Development, NIH PHEO/PGL registry were included in the present study. The number of males in the present study was significantly higher than the number of females (P = 0.008), but these two groups did not differ in any of these following parameters: size of the primary tumor (P = 0.13); percentage of patients with synchronous metastases (P = 0.36); time to metastasis (P = 0.94); or overall survival (P = 0.36) (Figure 1A).

Eighty-nine patients presented with PGL and 17 with PHEO (P < 0.001). The median ages at initial diagnosis of PGL or PHEO were 29 and 31 years, respectively. The age at diagnosis did not differ for different tumor sizes (Table 1). However, tumors with a smaller diameter were diagnosed significantly more often in the recent years (P = 0.043) (Table 1). The median size of all primary tumors was 6 cm. The median sizes of the primary PGLs and PHEOs were 6 cm and 8 cm, respectively (P = 0.023). Despite differences in the median sizes of the primary PGLs and PHEOs, the survival of patients diagnosed with either PGL or PHEO was not significantly different (P = 0.099) (Figure 1B). All the patients considered survival failures died due to metastatic PHEO/PGL.

**Table 1:** Comparison of the 4 tumor size groups

	**Type of tumor**	**Size of primary tumor**				**P-value**
		**<= 4 cm**	**4 - 6 cm**	**6 - 9 cm**	**> 9 cm**	
**No. of patients by tumor size group [%]**	ALL	32 [30]	24 [23]	25 [24]	25 [24]	**0.034 (PGL vs. PHEO)**
	PGL	31 [35]	19 [21]	20 [22]	19 [21]	
	PHEO	1 [6]	5 [29]	5 [29]	6 [35]	
**Probability of 5-year survival [%]**	ALL	94.1	95	83.4	88	**0.16**
	PGL	93.8	93.8	85.0	89.5	**0.26**
	PHEO	100	100	75.0	83.3	**0.39**
**Years to death**^**(median)**^	ALL	>55	>25	12	20	**0.035**
	PGL	>55	>25	12	20	**0.030**
	PHEO	>5	9	8	17	**0.58**
**Survival Hazard Ratio**	ALL	1	4.6	12.21	5.82	
**No. of deceased patients [%]**	ALL	1 [3.13]	6 [25]	10 [40]	6 [24]	
**Median age at diagnosis**	ALL	32	25	30	31	**0.44**
**Median year of diagnosis**	ALL	2007	2003	2004	2003	**0.043**
**Years to metastases (median)**	ALL	8	4	3	1	**0.0008**
**Probability of 5-year “metastases-free interval” [%]**	ALL	66.2	34.6	25.1	19.2	**0.0002**
**Probability of 10-year “metastases-free interval” [%]**	ALL	34.0	12.4	16.8	19.2	**0.004**

### Development of metastatic disease

Seventy-seven out of our 106 patients (72.6%) were diagnosed with metastatic disease over the course of their disease in the present study. Twenty-eight patients (26.4%) developed metastatic disease at the time of their primary tumor diagnosis or within six months (synchronous metastases); their median age at the initial diagnosis was 31.5 years; the median size of their primary tumor was 7.5 cm. Of the 78 patients not presenting with synchronous metastases, 49 (46.2% of total) went on to develop metachronous metastases, within the median time to metastases of 5 years; their median age at the initial diagnosis was 30 years; their median size of their primary tumors was 7.0 cm. For the remaining 29 (27.4% of total) patients who never developed metastatic disease, the median age at initial diagnosis was 29 years; their median size of the primary tumor was only 3.8 cm. The size of the primary tumors was found to be highly statistically different among the 3 groups described above (ANOVA, P < 0.001).

Patients with *SDHB*-related PHEO or PGL did not differ in the time to the development of metastasis (P = 0.54), and the probability of a 5-year metastasis-free interval among those without synchronous metastasis was similar (48.2% for PGL and 55.0% for PHEO).As described in Material and Methods, we used an ROC curve to establish an optimal cut-off size of the primary tumor in regards to the development of any metastases; the primary tumor size of 4.5 cm was the size that maximized the sum of the sensitivity and specificity to develop metastatic disease. The patients with primary tumors <4.5 cm had a median time to develop metachronous metastases of 8 years (CI 95%, 3 years to infinity), while those with larger tumors (≥4.5 cm) had a median time of only 2 years (CI 95%, 1 to 4 years; P = 0.003) (Figure 1C).

### Effect of metastatic disease development on the survival rate

Regardless of the presence of PHEO or PGL or their primary size, the overall 5-year survival probability of patients with any type of metastases was 75.7% (CI 95%; 63%-84%). The 5-year survival probability from the initial diagnosis for patients who presented with synchronous metastases was 74.5%; for patients not presenting with synchronous metastases, it was 96.4% (P = 0.006). However, once the patient was diagnosed with metastatic disease, there was no significant difference in their 5-year survival from the time of that diagnosis (74.5% for patients with synchronous metastases and 77.0% for metachronous metastases; P = 0.42).

As shown in Table 2, the presence of synchronous metastases did not have a significant effect on the survival of patients with the smaller tumors <5.5 cm (P = 0.63), but it had a highly significant effect on the survival of patients with larger tumors ≥5.5 cm (P = 0.0003). Specifically, the patients with the larger tumors (≥5.5 cm) and synchronous metastases had a 5-year survival probability of 65.8%, while patients with the same size primary tumors, but without synchronous metastases, had a 5-year survival probability of 97.1%. Interestingly, the above findings were observed only in PGLs, not in PHEOs (Table 2).

**Table 2:** 5-year survival probability of patients with tumors by tumor type (PHEO vs. PGL) and tumor size based on the presence of synchronous metastasis

	**Without synchronous metastases (n = 78)**	**Synchronous metastases (n = 28)**	**5-year/overall survival p-value**
**All tumors by type**			
PGL (n = 89)	97.9% (n = 66)	73.2% (n = 23)	0.0002/0.001
PHEO (n = 17)	88.9% (n = 12)	80.0% (n = 5)	0.56/0.94
5-year/overall survival p-value	0.19/0.012	0.79/0.89	---
**All tumors by size**			
<5.5 cm (n = 44)	95.2% (n = 37)	100% (n = 7)	0.63/0.41
> = 5.5 cm (n = 62)	97.1% (n = 41)	65.8% (n = 21)	0.0003/0.0028
5-year/overall survival p-value	0.71/0.18	0.09/0.021	---
**PGLs by size**			
<5.5 cm (n = 41)	94.7% (n = 34)	100% (n = 7)	0.61/0.40
> = 5.5 cm (n = 48)	100% (n = 32)	60.9% (n = 16)	0.0001/0.0002
5-year/overall survival p-value	0.22/0.57	0.068/0.014	---
**PHEOs by size**			
<5.5 cm (n = 3)	100% (n = 3)	N/A	N/A
> = 5.5 cm (n = 14)	85.7% (n = 9)	80.0% (n = 5)	0.68/0.86
5-year/overall survival p-value	0.59/0.38	N/A	---

### Effect of primary tumor size on survival time

As mentioned previously, the primary tumor cut-off size for predicting development of metastases was 4.5 cm. We observed that all the patients who died due to disease suffered from metastatic PHEO/PGL spread. In order to provide the most precise and clinically relevant information regarding the outcome (survival time) of these patients, another ROC analysis was performed, which established a 5.5 cm diameter as the optimal cutoff size for predicting survival within the present cohort. Accordingly, for survival analyses, patients were divided into two groups: <5.5 cm and ≥5.5 cm. When we analyzed the effect of primary tumor size on the survival time of all patients with *SDHB*, we found that patients with primary tumors <5.5 cm had significantly longer overall survival than patients with ≥5.5 tumors (P = 0.008) (Figure 1C). When the two tumor types were analyzed separately, the effect of size was highly significant in PGLs (P = 0.012), but was not significant in PHEOs (P = 0.39). However, this size-based survival difference in PGLs was present only for overall survival; for survival during the initial 5-year interval the difference between patients with smaller and larger tumors was not significant (P = 0.12).

### Effect of *SDHB* mutation type

In the present study, patients had a variety of *SDHB* mutation types: 13 had deletions (PGLs 11/PHEOs 2), 7 had frame-shift mutations (PGLs 4/PHEOs 3), 41 had missense mutations (PGLs 33/PHEOs 8), 24 had nonsense mutations (PGLs 20/PHEOs 4), and 21 had splice site mutations (PGLs 17/PHEOs 4). We did not find any significant differences in tumor size or survival time among different *SDHB* mutation types (P = 0.74 for size, P = 0.61 for survival time). The smallest tumors were found in the group of patients with frame-shift mutations, the largest tumors in patients with nonsense mutations.

### Size and age as independent predictors of survival and metastatic disease

Cox regression models, including interaction terms (as described in the Methods section), were used to evaluate tumor size and patient age at primary diagnosis as separate survival predictors. In none of the analyses was the interaction term between these two factors significant and hence it was not included in the final analyses. In the PGL group the size of the primary tumor and the age at initial diagnosis were found to be strong independent predictors of patient survival (P = 0.007 and P < 0.001, respectively). In the PGL group, patients diagnosed at a younger age had better performance, as did patients with smaller tumors, defined by the ordered categories as described in the Methods section. Furthermore, in the PGL group age at diagnosis did not predict the time to development of metastases (P = 0.51), but the size of the primary tumor did (P = 0.003), with patients having larger tumors more likely to develop metastatic disease.

In the PHEO group age at diagnosis was an independent predictor of patient survival (P = 0.041) but not for the development of metastases (P = 0.21), similar to the situation in the PGL group. In PHEO, as for PGL, younger patients had the better performance. However, unlike the situation in PGL patients, in PHEO tumor size was not found to be an independent predictor of either patient survival (P = 0.49) or the development of metastases (P = 0.65).

## Discussion

In the present study of 106 patients with pathogenic *SDHB* germline mutations, we found that the size of the primary tumor is an age-independent predictor of patient survival and metastases development in PGL. In both PHEO and PGL, age at diagnosis was found to be a size-independent predictor of patient survival. Furthermore, the development of synchronous metastases significantly affected 5-year and overall survival in patients with PGL. Patients with PHEO had possibly worse, though it did not reach statistical significance, overall survival than those with PGL (P = 0.099); their survival was not affected by the size of the primary tumor or by synchronous metastases. We did not find a significant difference in metastases development or patient survival between males and females or among specific *SDHB* mutations.

The importance of the primary tumor size for patient prognosis in general oncology is well established, as manifested by the use of the TNM classification [21]. Thus, previous studies have already agreed that the size of the primary tumor is an important predictor for patient survival and for the metastatic potential of PHEO/PGL, but none of these studies examined whether this finding was true of tumors with specific genetic backgrounds [12,17,19]. This study extends this knowledge, due to its unique design, to *SDHB*-related PHEO/PGL.

Evaluating two different parameters (survival and time to metastases) using ROC, we determined 2 optimal tumor size cut-offs for our study in order to provide more precise and clinically relevant information about these aggressive tumors. We established the optimal primary tumor size cut-off for the development of metastases as 4.5 cm; patients with smaller tumors developed metastases significantly later than patients with larger tumors. For the survival analyses, the patient cohort was separated into two groups, smaller tumors (<5.5 cm) and larger ones (≥5.5 cm), using a cut-point that was close to the median tumor size of PGLs, by far the larger of the two disease groups. Patients with smaller tumors had significantly better survival than patients with larger tumors, without separating PGL/PHEO. These two different cut-offs clinically show how the development of metastatic disease precedes disease mortality.

Alternatively the patients were divided into four groups according to the size of the primary tumor (≤4 cm, 4–6 cm, 6–9 cm, >9 cm); this division we considered clinically relevant and also resulted in groups of almost equal size (32/24/25/25). The percentages with synchronous metastases in these four groups were 22%, 13%, 28%, and 44%, respectively (trend P = 0.049, by exact test for contingency tables with ordered columns) (Table 1).

Studies evaluating exclusively metastatic PHEO/PGL patients have found that about one-third of these patients harbor pathogenic *SDHB* mutations [4,9]; however, the reported metastatic rate in *SDHB*-related PHEO/PGL varies dramatically [22]. In the present study we found it to be high, with 72.6% of the patients developing metastases over the course of the study, which is similar to the 71.7% previously reported in the study by Amar et al. [9]. Consequently, the presence of an *SDHB* mutation was found to be an independent predictor of PHEO/PGL malignant behavior [5]. Typical metastatic sites of *SDHB*-related PHEO/PGL include the bones, lungs, lymph nodes, and liver; multiple metastases are also possible [5].

Furthermore, in childhood and adolescence the incidence of PHEO/PGL is rare; however, when metastatic disease is diagnosed in these age groups, these patients have a high probability of having *SDHB* mutations [23]. The present study showed better performance of younger patients and established the age at diagnosis as a survival predictor in patients with *SDHB* PHEO/PGL. Improved survival of younger patients was already reported in a study by Amar et al., but did not reach significance as an independent predictor [5].

The previously reported ratio between synchronous and metachronous metastases in various PHEOs/PGLs was almost equal (51%/49%) [19]. In our study with only *SDHB*-related tumors, we observed predominantly metachronous rather than synchronous metastases (64%/36%), which could be due to the relatively long follow-up periods for our patients. We did not find a significant difference in the 5-year survival between patients after the diagnosis of synchronous or metachronous metastatic disease, which suggest that tumors with synchronous metastases are not inherently more aggressive.

There is still disagreement on the relative survival of patients with PHEO and PGL. A previous study, which did not classify the tumors based on genetic background, found overall survival to be significantly shorter in patients with PGLs than with PHEOs [19]. A different study, which also did not consider the genetic background of the disease and included only metastatic PHEO/PGL, reported better survival of PGLs [24]. Our study, including only patients with *SDHB*-related tumors, found that patients with PGLs had a better overall survival than patients with PHEOs. However the difference was not statistically significant, quite possibly due to the small number of PHEOs as opposed to PGLs in these patients with *SDHB* mutations (Figure 1A). While elucidating other possible differences between *SDHB*-related PHEO and PGL, we demonstrated that the size of the primary tumor for patients with PHEOs seems to be less important for patient survival than for PGL. We initially attributed this to the selected cut-off (5.5 cm), which was more representative of the size of primary PGLs (and divided them almost evenly) and divided the PHEOs quite unevenly. However, when we used 8 cm as a cutoff, the median size of PHEO primary tumors, there was again no survival difference in PHEOs between those with small vs. large tumors (P = 0.81) Significantly larger tumors in patients with PHEO than with PGL have been reported previously [24].

Our analysis clearly shows that the size of the primary tumor in PGLs predicts the development of metastatic disease and also affects patient survival, while the age at diagnosis predicts patient survival but not the development of metastases. In contrast, in the limited number of *SDHB*-mutated PHEOs, we found that the age at diagnosis is an important factor for survival, but the size of the primary tumor is not. The lack of an observed significant effect of primary tumor size in PHEOs is not in agreement with the previous observation of Zelinka et al. [17], who evaluated a larger sample of metastatic PHEOs, but did not focus on the genetic background of the PHEOs included in that study, both of which could explain this difference.

Given the status of the NIH PHEO/PGL program as a national and international referral center, our patient population is typically made up of clinically more severe and complicated cases, usually due to patients with underlying genetic backgrounds, which could possibly lead to some referral bias. Therefore, we were not able to establish a similarly sized cohort of apparently sporadic patients that would allow us to investigate whether these tumors would behave similarly to *SDHB*-related PHEO/PGL and to elucidate how exactly the presence of an *SDHB* mutation would affect the development of metastatic disease and patient survival when the size of the primary tumor is considered and compared to other PHEO/PGL types. Since the incidence of *SDHB*-related PHEOs is very low compared to *SDHB*-related PGLs, it was very difficult to reliably compare these two groups, and other significant findings might become more apparent if a larger number of PHEOs were available. Similarly, it was very difficult to evaluate the effect of size on metastases development in *SDHB*-related PHEOs, because there were only 17 such patients and only 4 of these (23.5%) did not develop any metastases. Of the 89 patients with PGL, 25 (28.09%) did not develop metastases during the follow-up period of this study. The PGL patient cohort also included patients with head and neck PGLs, because our main goal was to fully evaluate the *SDHB* patient population; however, a bigger patient cohort separating those patients from other PGL patients might provide further interesting results.

## Conclusions

In summary, this unique study of patients with *SDHB*-related PHEO/PGL showed that the age at the primary diagnosis as well as the size of the primary tumor are two important independent prognostic factors. We did not find any difference based on the sex of the patient, but we did observe certain differences between *SDHB*-related PGLs and PHEOs. This data strongly supports our recommendations that all carriers with *SDHB* mutations should undergo early and regular evaluations to detect tumor(s) at an early stage to achieve the best clinical outcome with regards to their survival.

## Competing interests

The authors declare that they have no competing interests.

## Authors’ contributions

JS and KP designed the study. JS, VM, ZF, and KP wrote the initial draft of the manuscript. RW performed the statistical analyses. All authors assisted in the data collection and approved the final manuscript before submission.

## Pre-publication history

The pre-publication history for this paper can be accessed here:

http://www.biomedcentral.com/1471-2407/14/523/prepub

## Supplementary Material

Additional file 1: Figure S1 ROC analyses showing establishment of 2 cut-offs for the present study. For survival analyses: AUC = 0.663 (P < 0.0001), optimal cutpoint = 5.5 cm, sensitivity = 87.0%, specificity = 49.4%. For analyses of metastatic development: AUC = 0.782 (P < 0.0001), optimal cutpoint = 4.5 cm, sensitivity = 80.5%, specificity = 69.0%. Click here for file
