# Supplementary figures and images for: The size of the primary tumor and age at initial diagnosis are independent predictors of the metastatic behavior and survival of patients with SDHB-related pheochromocytoma and paraganglioma: a retrospective cohort study
Source: BMC Cancer. 2014 Jul 21;14:523. doi: 10.1186/1471-2407-14-523 (PMC4223758; doi:10.1186/1471-2407-14-523)

ROC: Survival or Metastases by Size

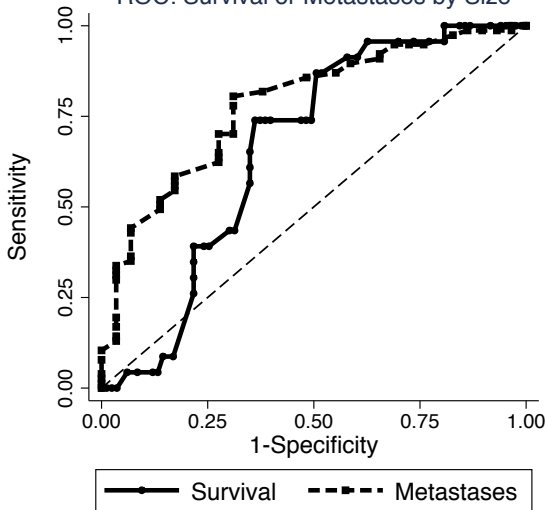

Supplement: Additional file 1: Figure S1 — ROC analyses showing establishment of 2 cut-offs for the present study. For survival analyses: AUC = 0.663 (P < 0.0001), optimal cutpoint = 5.5 cm, sensitivity = 87.0%, specificity = 49.4%. For analyses of metastatic development: AUC = 0.782 (P < 0.0001), optimal cutpoint = 4.5 cm, sensitivity = 80.5%, specificity = 69.0%. [file 1471-2407-14-523-S1.pdf]
